# A randomized, double‐blind, placebo‐ and positive‐controlled crossover study of the effects of durlobactam on cardiac repolarization in healthy subjects

**DOI:** 10.1111/cts.12991

**Published:** 2021-05-02

**Authors:** John O’Donnell, Kathleen Maloney, Melissa Steidler, Royce Morrison, Robin Isaacs

**Affiliations:** ^1^ Entasis Therapeutics Waltham Massachusetts USA; ^2^ Johnson & Johnson Spring House Pennsylvania USA; ^3^ Pharmaron Clinical Pharmacology Center Baltimore Maryland USA

## Abstract

Durlobactam (formerly ETX2514) is a diazabicyclooctane β‐lactamase inhibitor that inhibits class A, C, and D β‐lactamases. Sulbactam combined with durlobactam has in vitro and in vivo activity against *Acinetobacter baumannii* including carbapenem‐ and colistin‐resistant isolates and is being developed for treating serious infections due to *A*.* baumannii*. The effect of a single supratherapeutic dose of durlobactam on the heart rate corrected QT interval (QTc) was evaluated in healthy subjects in a placebo‐ and active‐controlled, single‐infusion, three‐way crossover study. Subjects were randomized to 1 of 6 sequences that included a single 3‐h i.v. infusion of durlobactam 4 g (supratherapeutic dose), a single 3‐h i.v. infusion of placebo, and a single 3‐h i.v. infusion of placebo plus a single oral dose of moxifloxacin 400 mg given open‐label at the end of the i.v. infusion. In each treatment period, Holter electrocardiogram (ECG) measurements were obtained from predose through 24 h post‐start of infusion. For the primary ECG end point, placebo‐corrected change‐from‐baseline corrected QT Fridericia’s formula (ΔΔQTcF), no significant change was observed with durlobactam. A concentration‐QT analysis demonstrated no significant effect of durlobactam on ECG parameters, including QT interval prolongation. Thus, durlobactam has a low risk for prolonging the QT interval and is unlikely to produce any proarrhythmic effects.

**Study Highlights**

**WHAT IS THE CURRENT KNOWLEDGE ON THE TOPIC?**

Drug‐induced prolongation of the QT interval has the potential to cause severe, potentially fatal ventricular arrhythmias. A number of antimicrobial agents, including fluoroquinolones and macrolides, are associated with a low, but clinically significant increased risk of QT prolongation.

**WHAT QUESTION DID THIS STUDY ADDRESS?**

This study evaluated the effect of a single supratherapeutic dose of durlobactam on the heart rate corrected QT interval in healthy subjects to determine if there were any potentials for proarrhythmic effects.

**WHAT DOES THIS STUDY ADD TO OUR KNOWLEDGE?**

This study found that durlobactam had a low risk for prolonging the QT interval and is unlikely to produce any proarrhythmic effects.

**HOW MIGHT THIS CHANGE CLINICAL PHARMACOLOGY OR TRANSLATIONAL SCIENCE?**

Because durlobactam had a low risk for prolonging the QT interval alone and when co‐administered with sulbactam, clinicians should be confident in administering the combination without risk for proarrhythmic effects.

## INTRODUCTION

Durlobactam (formerly ETX2514) is a diazabicyclooctane β‐lactamase inhibitor that inhibits class A, C, and D β‐lactamases.^1^, ^2^, ^3^ A combination of the β‐lactamase inhibitor, sulbactam, and durlobactam demonstrates in vitro and in vivo activity against *Acinetobacter baumannii* isolates, including carbapenem‐ and colistin‐resistant isolates.^3^, ^4^, ^5^, ^6^, ^7^ In phase I clinical studies among healthy subjects, the pharmacokinetic (PK) profile of durlobactam alone and in combination with sulbactam was evaluated after single and multiple‐ascending i.v. doses and demonstrated a dose‐proportional increase in exposure and potentially therapeutic concentrations in pulmonary tissues with no drug interactions with sulbactam, cilastatin, and imipenem.^8^, ^9^, ^10^ The sulbactam‐durlobactam combination was generally safe and well‐tolerated in a phase II study of hospitalized patients with complicated urinary tract infections.^11^ Sulbactam‐durlobactam is being developed for the treatment of infections caused by *Acinetobacter baumannii*‐*calcoaceticus* Complex, including multidrug resistant and carbapenem‐resistant isolates.

Drug‐induced prolongation of the QT interval has the potential to cause severe, potentially fatal ventricular arrhythmias.^12^ A number of antimicrobial agents, including fluoroquinolones and macrolides, are associated with a low, but clinically significant increased risk of QT prolongation.^13^, ^14^, ^15^, ^16^ As a consequence, most new chemical entities undergo rigorous evaluation of QT interval prolongation to assess the potential to delay cardiac repolarization that leads to the development of ventricular arrhythmias (i.e., torsade de pointes) and may result in sudden death.^17^, ^18^

The objective of this study was to evaluate the effect of a single supratherapeutic dose of durlobactam on the heart rate (HR) corrected QT interval (QTc) in healthy volunteers. Secondary objectives were to evaluate the effects of a supratherapeutic dose of durlobactam on other electrocardiogram (ECG) parameters and the PKs, safety, and tolerability of durlobactam. The effects on QT interval were not evaluated for sulbactam, which has undergone extensive clinical use as the combination product Unasyn (sulbactam + ampicillin) and has not shown any pro‐arrhythmic effects. Lower peak plasma concentration afforded with a 3 h infusion of sulbactam‐durlobactam and the lack of drug‐drug interactions also suggests lower peak sulbactam concentrations will be realized clinically with sulbactam‐durlobactam relative to administration of sulbactam + ampicillin at approved dose levels.

## METHODS

The study was conducted in accordance with the US Code of Federal Regulations and ethical principles of the Declaration of Helsinki, Good Clinical Practices, and the International Council for Harmonization guidelines. The study protocol and all amendments were reviewed by the institutional review board for the study center (IntegReview IRB, Austin, TX). Informed consent was obtained from each subject in writing before randomization.

### Study design

This was a partially double‐blind study in healthy adult subjects, which was conducted as a placebo‐ and active‐controlled, single‐infusion, three‐way crossover study. Eligible subjects were randomized to 1 of 3 treatments administered in 1 of 6 randomized sequences: a single 3‐hour i.v. infusion of durlobactam 4 g (supratherapeutic dose), a single 3‐h i.v. infusion of placebo, and a single 3‐h i.v. infusion of placebo with a single oral dose of moxifloxacin 400 mg given open‐label at the end of the i.v. infusion. Study treatments were administered in the fasted state on day 1 of each treatment period. A 7 (±2) day washout period occurred between successive treatments. Subjects, investigator, and sponsor were blinded to durlobactam and placebo treatment assignment. Moxifloxacin was administered open‐label.

In each treatment period, Holter ECG measurements were obtained from predose through 24 h post‐start of infusion. Variables associated with the primary ECG end point, placebo‐corrected change‐from‐baseline QTcF (ΔΔQTcF), were computed from means of measurements made on multiple replicate ECGs extracted at scheduled timepoints from Holter 12‐lead ECG data. Holter 12‐lead ECGs were read centrally (ERT, Rochester, NY). ECG end points included change‐from‐baseline HR, QTcF, PR, and QRS (ΔHR, ΔQTcF, ΔPR, and ΔQRS); placebo‐corrected change‐from‐baseline HR, PR, and QRS (ΔΔHR, ΔΔPR, and ΔΔQRS); categorical outliers for QTcF, HR, PR, and QRS; and frequency of treatment‐emergent changes of T‐wave morphology and U‐wave presence.

### Subject selection

Men or women age 18–55 years and a body mass index greater than or equal to 18.0 and less than or equal to 30.0 kg/m^2^ at screening were eligible if they were medically healthy with no clinically significant abnormalities on medical history, physical examination, laboratory testing, vital signs, or ECG. At screening, subjects were required to have a normal sinus rhythm, HR of 45 to 100 beats/min, QTcF interval less than 450 ms, QRS interval less than or equal to 110 ms, and PR interval less than or equal to 220 ms, as well as a supine blood pressure between 90/40 and 140/90 mm Hg. Women of childbearing potential (i.e., not postmenopausal or surgically sterilized) must have a negative serum pregnancy test before randomization. Participating heterosexual women of childbearing potential must be willing to consistently use two highly effective methods of contraception (i.e., condom with spermicide, combined oral contraceptive, implant, injectable, indwelling intrauterine device, or a vasectomized partner) from screening until at least 30 days after administration of the last dose of study drug.

Subjects were excluded for a history of ventricular pre‐excitation syndrome (Wolff‐Parkinson White syndrome); arrhythmia, or history of arrhythmia requiring medical intervention; risk factors for torsade de pointes (e.g., heart failure, cardiomyopathy, or family history of long QT syndrome); or sick sinus syndrome, second or third degree atrioventricular block, myocardial infarction, pulmonary congestion, symptomatic or significant cardiac arrhythmia, prolonged QTcF interval, or conduction abnormalities.

### Study assessments

At screening, all subjects underwent a comprehensive physical examination and provided a medical history; vital signs (blood pressure and HR, respiratory rate, and body temperature), safety 12‐lead ECG, and routine laboratory tests (hematology, chemistry, and urinalysis) were obtained. Serology tests, urine drug screening, and pregnancy tests (women only) were performed.

Cardiodynamic assessment was performed for all 3 treatment periods, from predose through 24 h post‐start of infusion. During each treatment period, 12‐lead, 24‐h Holter ECGs were continuously recorded and evaluated by a central ECG laboratory. All 12‐lead ECG data were extracted from continuous recordings predose (−45, −30, and −15 min), 1.5 h (during the infusion), 3 h (end of infusion), and 3.25, 3.5, 4, 5, 6, 7, 8, 12, and 24 h post‐start of infusion, for a total of 14 timepoints.

Blood samples were obtained at −45, −30, and −15 min prior to the infusion, at 1.5 during the infusion, and at 3, 3.25, 3.5, 4, 5, 6, 7, 8, and 24 h after the infusion. Plasma concentrations less than the lower limit of quantification were reported as 0. Plasma concentrations of durlobactam were determined by validated liquid chromatography with tandem mass spectrometry (LC‐MS/MS) assays operated in the negative ion mode (method number M8351072B) performed at Covance (Salt Lake City, UT). For durlobactam, the quantitation range was 5–5000 ng/ml. Moxifloxacin plasma concentrations were determined by LC‐MS/MS operated in the positive ion mode (method number 180M001.01) performed at Pharmaron ABS (Germantown, PA). The quantitation range was 1–1000 ng/ml.

PK parameters included maximum plasma concentration (C_max_), time to maximum concentration (T_max_), terminal elimination half‐life calculated, area under the concentration‐time curve from time 0 to infinity (AUC_0‐inf_), AUC time 0 to last non 0 value (AUC_0‐last_), and AUC from time 0 to 24 h (AUC_0‐24_), volume of distribution (V_z_), and total body clearance (CL). PK parameters were determined with noncompartmental analysis using Phoenix WinNonlin (version 7.0 or higher), and PK parameters were compared with SAS version 9.3 or higher.

Safety was assessed from vital signs (systolic/diastolic blood pressure, HR, respiratory rate, and body temperature), physical examination, clinical laboratory tests (serum chemistry, hematology, and urinalysis), 12‐lead safety ECGs, and adverse events (AEs).

### Statistical analysis

Thirty‐two subjects were randomized to provide a sample size of at least 24 evaluable subjects with data from all treatment periods. A sample size of 24 evaluable subjects would provide greater than 90% power to exclude that durlobactam caused more than a 10 ms QTc effect at the observed geometric mean C_max_ based on the upper bound of the 2‐sided 90% confidence interval (CI) of ΔΔQTcF at this concentration. The calculation assumed an underlying effect of durlobactam of 3 ms and a SD of the ΔQTcF of 8 ms. In addition to the evaluation through modeling and simulation, the sample size was estimated approximately using a simple paired *t*‐test for equivalence. Under the assumption that the QTcF effect was 3 ms for durlobactam and 0 ms for placebo with an SD of ΔQTcF of 8 ms for each treatment, and that “no effect” was able to be established if the 90% CI of placebo‐corrected ΔQTcF was lower than 10 ms, 24 subjects provided greater than 90% power with a 1‐sided alpha of 5% in the paired *t*‐test. This estimation was done using the paired *t*‐test for equivalence of means in R version 3.2.5.

Data were analyzed using SAS, version 9.3 or higher (SAS Institute, Cary, NC, SAS System). Data were summarized with descriptive statistics for continuous variables and number of subjects, mean, SD, minimum, median, and maximum. The SE and 90% CI were included if applicable. For categorical variables, descriptive statistics included counts and percentages.

### Concentration‐QTc analysis

The relationship between durlobactam plasma concentration and change‐from‐baseline QTcF (ΔQTcF) was investigated by a linear mixed‐effects modeling approach with a treatment effect‐specific intercept. From the model, the slope (i.e., the regression parameter for the concentration) and the treatment effect‐specific intercept (defined as the difference between active and placebo) were estimated together with the 2‐sided 90% CI. The geometric mean of the individual C_max_ values for subjects on the active dose were determined. The predicted effect and its 2‐sided 90% CI for placebo corrected change‐from‐baseline ΔΔQTcF (i.e., slope estimate × concentration + treatment effect‐specific intercept) at this geometric mean C_max_ of durlobactam were obtained. If the upper bound of the 2‐sided 90% CI of the predicted effect of ΔΔQTcF at clinically relevant plasma levels of durlobactam was below 10 ms, it was to be concluded that durlobactam does not cause clinically relevant QTc prolongation. Assay sensitivity was evaluated by concentration QTc (C‐QTc) analysis of the effect on ΔΔQTcF of moxifloxacin using the same model. Assay sensitivity was deemed met if the slope of the C‐QTc relationship was statistically significant at the 10% level in a 2‐sided test and the predicted QTcF effect (i.e., the lower bound of the 2‐sided 90% CI of ΔΔQTcF) was above 5 msec at the observed geometric mean C_max_ of 400 mg moxifloxacin.

## RESULTS

### Disposition and baseline characteristics

Thirty‐two subjects were enrolled in the study, and 31 received a dose of durlobactam. Two subjects discontinued, one for voluntary withdrawal of consent following durlobactam dosing and the other lost to follow‐up following moxifloxacin treatment. Subject characteristics were similar across treatment sequences at baseline (Table 1).

**Table 1:** Baseline characteristics of study subjects

Characteristic	Subjects (*N* = 32)
Age, y^a^	34.2 ± 9.5
Age range, y	20–54
Male, *n* (%)	17 (53.1)
Hispanic or Latino, *n* (%)	7 (21.9)
Race, *n* (%)	
White	10 (31.2)
Black or African American	22 (68.8)
Body weight, kg^a^	73.9 ± 10.3
Body mass index, kg/m^a^	25.4 ± 2.9

^a^ Mean ± SD.

### Pharmacokinetic analysis

All subjects had quantifiable plasma concentrations of durlobactam starting at 1.5 h post‐start‐of‐infusion (first post‐start‐of‐infusion sampling timepoint), and concentrations remained quantifiable out to the last sampling time of 24‐h post‐start‐of‐infusion in all subjects across all 3 periods. Twenty‐two of 31 subjects had quantifiable plasma concentrations of moxifloxacin starting at 3.25 h post‐start‐of‐infusion (first PK sampling timepoint post‐dose for moxifloxacin). All subjects had quantifiable moxifloxacin concentrations starting at 4 h post‐start‐of‐infusion and remained quantifiable out to the last sampling time of 24 h post‐start‐of‐infusion in all subjects across all 3 periods.

**Figure 1:**
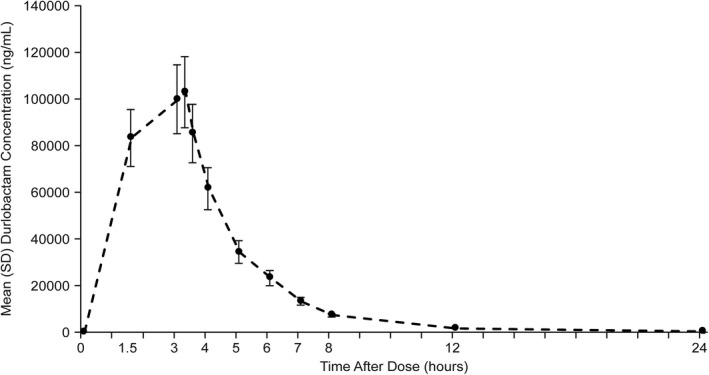
Concentration‐time curve for plasma durlobactam after a 4 g dose, *n* = 31 (pharmacokinetic [PK] population)

In periods 1 through 3 combined, following a single 3‐hour i.v. infusion of 4 g durlobactam, plasma concentrations of durlobactam reached a peak by ~ 3 h after the start of the infusion and rapidly declined with a mean apparent half‐life of 2.3 h (Figure 1). The mean C_max_ of durlobactam was 107,941.9 ng/ml, mean AUC_0‐24 h_ was 413,100.9 h*ng/ml, and mean AUC_0‐inf_ was 413,345.9 h*ng/ml (Table 2). The mean CL of durlobactam was ~ 9.9 L/h and a mean V_z_ of 33.608 L.

**Table 2:** Pharmacokinetic results after a single 4 g dose of durlobactam in healthy subjects (*n* = 31)

	Mean ± SD	Coefficient of variation (%)
C_max_, ng/ml	107,942 ± 19,946	18.5
T_max_, h	3.1 ± 0.32	10.6
AUC_0‐24_, h*ng/ml	413,101 ± 83179	20.1
AUC_0‐inf_, h*ng/ml	413,346 ± 83177	20.1
CL, L/h	9.9 ± 1.4	14.0
V_z_, L	33.6 ± 5.2	15.6

Abbreviations: AUC_0‐24_, area under the concentration‐time curve from time 0 to 24 h; AUC_0‐inf_, area under the concentration‐time curve from time 0 to infinity; CL, total body clearance; C_max_, maximum plasma concentration, T_max_, time to maximum concentration; *V*_z_, volume of distribution.

In periods 1 through 3 combined, following a single 400 mg dose of oral moxifloxacin, plasma concentrations of moxifloxacin reached a peak at ~ 1.9 h and declined with a mean apparent half‐life of 10.8 hours. The mean C_max_ of moxifloxacin was 2182.3 ng/ml and a mean AUC_0‐last_ was 22,638.6 h*ng/ml (Table 2).

### Cardiodynamic evaluation

**Figure 2:**
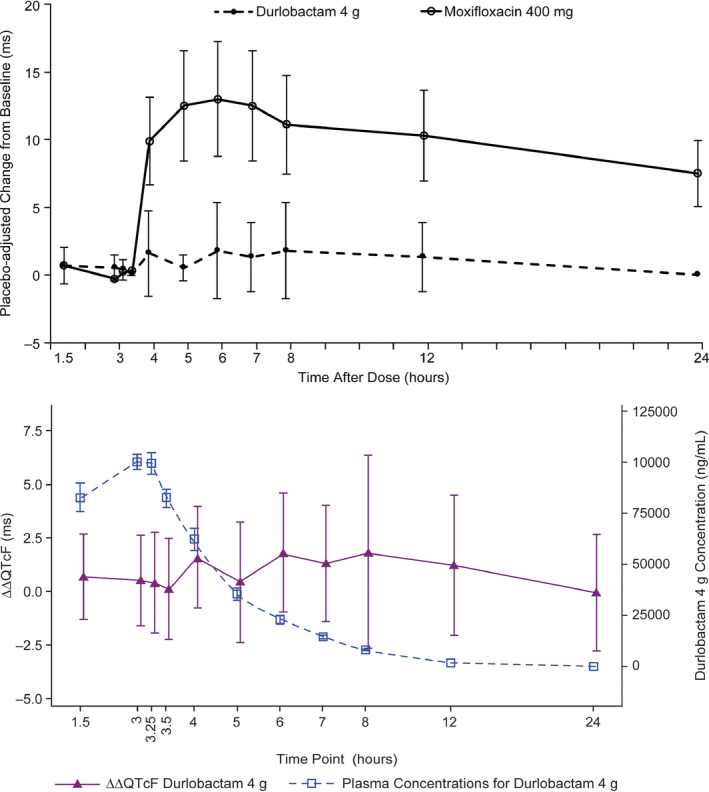
Placebo‐corrected change from baseline for QTcF (**ΔΔ**QTcF) across timepoints (LS mean and 90% confidence interval (CI) based on a linear mixed‐effects model). Bottom: Durlobactam plasma concentrations and **ΔΔ**QTcF over time (pharmacokinetic [PK]/QTc population)

A 4 g supratherapeutic dose of durlobactam had no clinically relevant effect on HR. Mean change from baseline HR (ΔHR) with durlobactam closely followed the pattern observed with placebo. Mean placebo‐corrected ΔHR (ΔΔHR) was small at post‐start‐of‐infusion timepoints, varying between −2.6 and −0.2 bpm at 3 and 4 h, respectively. Mean change from baseline QTcF (ΔQTcF) for durlobactam was similar to placebo at postdose timepoints (Table 3). Mean placebo‐corrected ΔQTcF (ΔΔQTcF) varied within a range between 0.0 ms (24 h post‐start‐of‐infusion) and 1.8 ms (6 and 8 h post‐start‐of‐infusion; Figure 2).

**Table 3:** Placebo‐corrected change from baseline QTcF (ddQTcF) at each timepoint (cardiodynamic population)

Timepoint postdose, h)	Durlobactam 4 g	Moxifloxacin 400 mg
1.5	0.7 ± 1.2 (−1.3, 2.7)	0.7 ± 1.2 (−1.4, 2.7)
3	0.5 ± 1.3 (−1.6, 2.7)	−0.3 ± 1.3 (−2.5, 1.8)
3.25	0.4 ± 1.4 (−2.0, 2.8)	0.2 ± 1.4 (−2.2, 2.6)
3.5	0.1 ± 1.4 (−2.3, 2.6)	0.3 ± 1.4 (−2.1, 2.7)
4	1.6 ± 1.4 (−0.8, 4.0)	9.9 ± 1.4 (7.5, 12.3)
5	0.5 ± 1.7 (−2.4, 3.3)	12.5 ± 1.7 (9.6, 15.3)
6	1.8 ± 1.7 (−1.0, 4.7)	13.0 ± 1.7 (10.2, 15.9)
7	1.3 ± 1.7 (−1.4, 4.1)	12.5 ± 1.7 (9.7, 15.2)
8	1.8 ± 2.8 (−2.8, 6.4)	11.1 ± 2.8 (6.4, 15.7)
12	1.3 ± 2.0 (2.1, 4.6)	10.3 ± 2.0 (7.0, 13.6)
24	0.0 ± 1.6 1.0 (−2.8, 2.7)	7.5 ± 1.6 (4.7, 10.2)

**Figure 3:**
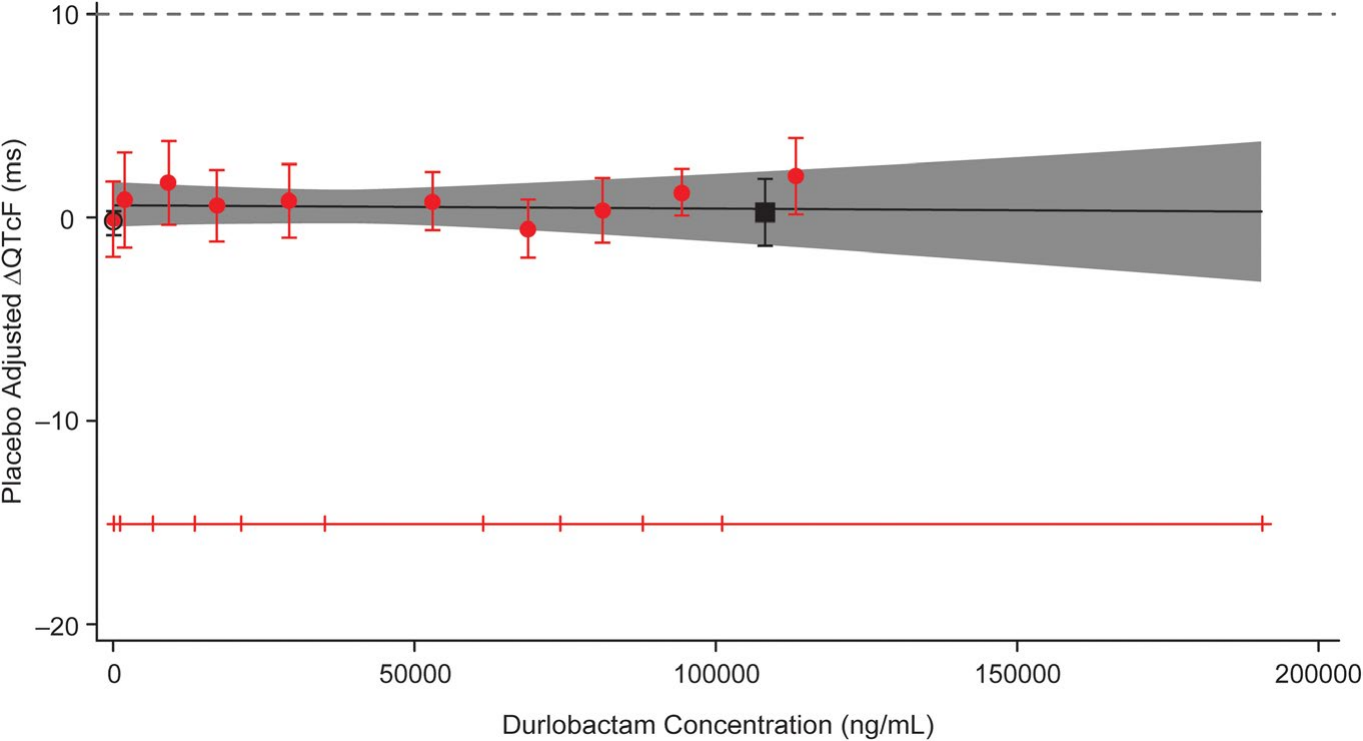
Model‐predicted corrected change from baseline for QTcF (ΔΔQTcF; mean and 90% confidence interval [CI]) and estimated placebo‐adjusted ΔQTcF (mean and 90% CI) across deciles of durlobactam plasma concentrations (top) and predicted ΔΔQTcF interval at geometric mean peak durlobactam concentrations after a 4 g dose (bottom) (pharmacokinetic [PK]/QTc population)

The hysteresis loop of durlobactam plasma concentration showed that ΔΔQTcF varied without relation to durlobactam plasma concentrations. In the concentration‐QTc analysis, a linear model with a treatment effect‐specific intercept was fitted for durlobactam plasma concentrations and was representative of the data (Figure 2). The estimated slope of the durlobactam plasma concentration‐QTc relationship was shallow and not statistically significant (−0.0000019 ms per ng/ml [90% CI: −0.0000232 to 0.0000194]) with a small and not statistically significant treatment effect‐specific intercept of 0.6 ms (Figure 3). The predicted QT effect (ΔΔQTcF) at the observed geometric mean durlobactam C_max_ (106,000 ng/ml) was 0.43 ms (90% CI: −1.38 to 2.23; Figure 3). Based on this concentration‐QTc analysis, an effect on ΔΔQTcF exceeding 10 ms was excluded up to approximate durlobactam concentrations of 190,000 ng/ml.

The mean placebo‐adjusted ΔQTcF (90% CI) within each moxifloxacin concentration decile and the model‐predicted mean ΔΔQTcF with 90% CI demonstrated that the predicted ΔΔQTcF values were close to the estimated placebo‐adjusted ΔQTcF across all plasma concentration levels except at the sixth and seventh deciles (Figure S1). The proposed model provided a reasonable representation of the relationship between placebo‐adjusted ΔQTcF and moxifloxacin concentrations.

Assay sensitivity was demonstrated using the same linear model for moxifloxacin. The treatment effect‐specific intercept was 0.4 ms, which was not statistically significant. The slope of the relationship was positive and statistically significant: 0.0065 ms per ng/ml (90% CI: 0.00544–0.00750). The mean placebo‐adjusted ΔQTcF (90% CI) within each moxifloxacin concentration decile and the model‐predicted mean ΔΔQTcF with 90% CI demonstrated that the predicted ΔΔQTcF values were close to the estimated placebo‐adjusted ΔQTcF across all plasma concentration levels except at sixth and seventh deciles (Figure S1). The proposed model provided a reasonable representation of the relationship between placebo‐adjusted ΔQTcF and moxifloxacin concentrations.

The predicted ΔΔQTcF at the geometric mean peak moxifloxacin concentration demonstrated that the lower bound of the 2‐sided CI of the predicted QT effect (13.99 ms [90% CI: 12.27 to 15.71]) at geometric mean peak moxifloxacin concentrations (2103.4 ng/mL) was above 5 ms demonstrating assay sensitivity (Figure S1).

### Categorical analysis of ECG parameters

An increase in the QTcF greter than 450 ms and less than or equal to 480 ms was reported in 1 (3.2%; 4 timepoints) subject with durlobactam, 2 (6.5%; 6 timepoints) with moxifloxacin, and 1 (3.3%; 7 timepoints) with placebo. A ΔQTcF greater than 30 ms and less than or equal to 60 ms was reported for 2 (6.5%) subjects with moxifloxacin.

### Safety and tolerability

The incidence of at least one AE was similar for each of the three treatment groups with six (19.4%), five (16.7%), and seven (22.6%) subjects in the durlobactam, placebo, and moxifloxacin groups, respectively, experiencing an AE. No serious AEs, discontinuation for AEs, or deaths occurred. The only AE occurring in more than one subject with durlobactam was fatigue (2 subjects, 6.5%).

No clinically relevant changes in clinical laboratory or vital signs were observed. No clinically significant safety ECG abnormalities were observed, including clinically relevant change in HR, RR interval, PR interval, QRS interval, QT interval, or QTcF interval between subjects. No trends were observed for change from baseline in HR, PR interval, QRS interval, QT interval, or QTcF interval over time. Abnormal values were varied and transient.

## DISCUSSION

At the 4 g i.v. dose administered over 3 h, durlobactam exhibited a PK profile that was consistent with results from previous studies.^8^, ^9^, ^11^ The results from the C‐QT analysis demonstrated no significant effect of durlobactam on ECG parameters including QT interval prolongation. An effect on the QTcF exceeding 10 ms was excluded at durlobactam plasma concentrations up to 190,000 ng/ml, which is over 3‐fold greater than therapeutic plasma concentrations at the planned dose of 1 g. Results with the positive control, moxifloxacin, confirmed assay sensitivity with this study design and test population. The safety and tolerability profile of durlobactam was consistent with previous studies,^8^, ^9^, ^11^ and no new safety signals were identified.

The US Food and Drug Administration requires an assessment of the effects of new drugs on QT interval prolongation as part of the clinical development program.^17^ Traditionally, this has required a thorough QT study. However, recent guidance from the International Council for Harmonization allows C‐QTc modeling as the primary analysis for assessing the risk of QTc interval prolongation with new drugs.^19^ As a consequence, a phase I clinical study in healthy subjects based on C‐QTc modeling provides an alternative to the traditional thorough QT study to exclude clinically relevant QTc effects. Previous studies have demonstrated the validity of a C‐QTc design for evaluating the effects of a drug on QT interval prolongation.^20^, ^21^

The dosing regimen of durlobactam being evaluated in a phase III study is sulbactam 1 g plus durlobactam 1 g administered by i.v. infusion over 3 h every 6 h. Thus, the durlobactam supratherapeutic dose used in this study was fourfold greater than the proposed clinical dose. Previous phase I studies with durlobactam demonstrated a PK profile consistent with the results reported here. After single i.v. doses of durlobactam, C_max_ was 26,900 ng/ml after a 1 g i.v. dose and 96,200 ng/ml after a 4 g i.v. dose.^8^ With a multiple dose regimen for 11 days, C_max_ after a 1 g i.v. dose was 28,100 ng/ml, indicating minimal accumulation with repeat dosing.^8^ A phase II study in patients with complicated urinary tract infection treated with durlobactam/sulbactam 1 g every 6 h for 7 days reported a C_max_ of 39,900 ng/ml.^11^ In a single dose study of healthy subjects with varying degrees of renal impairment, C_max_ was 27,000 ng/ml in those with normal renal function and ranged from 25500 to 33,300 ng/ml in those with mild, moderate, or severe renal impairment.^9^ Although a 500 mg dose of durlobactam was used for subjects with severe renal impairment in this study, linear regression analysis of dose normalized C_max_ exposure of durlobactam versus creatinine clearance (CLcr) suggested an estimate of 60,000 ng/ml for the severe renal impairment category (CLcr = 0–15 ml/min) corresponding to 70,000 ng/ml at steady‐state with a 1 g dose. Based on these considerations, durlobactam 4.0 g i.v. infused over 3 h was selected as the supratherapeutic dose for this study. The mean C_max_ of 107,942 ng/ml achieved in the present study with a 4 g dose represents approximately a 3.2‐fold and 1.5‐fold increase above the maximum C_max_ predicted in patients receiving a 1 g dose with normal renal function and those with severe renal impairment, respectively. Thus, the 4 g dose of durlobactam satisfies the regulatory requirement for a supratherapeutic dose to be used in a definitive study of QT effect. Sulbactam clinical experience, as used in a combination with ampicillin, has not suggested any proarrhythmic effects at the 1 g dose level of sulbactam (including renal function‐based dose adjustments). With the longer infusion of 3 h for sulbactam‐durlobactam and lack of drug‐drug interactions between the two compounds, higher concentrations of sulbactam are not anticipated relative to sulbactam + ampicillin use. Therefore, co‐administration of sulbactam was not evaluated in the present study.

In summary, based on the results of this study, durlobactam has a low risk for prolonging the QT interval and is unlikely to produce any proarrhythmic effect when administered with sulbactam, a drug shown to have a low risk for proarrhythmic effects.

## Disclosures

John O’Donnell, Kathleen Maloney, Melissa Steidler, and Robin Isaacs were paid employees of Entasis Therapeutics, Waltham, MA, at the time of this study. Royce Morrison was an investigator from Pharmaron compensated by Entasis Therapeutics in support of completing the study.

## AUTHOR CONTRIBUTIONS

J.O., R.I., and R.M. wrote the manuscript. J.O., R.M., and R.I. designed the research. K.M., M.S., and R.M. performed the research. J.O., K.M., M.S., R.M., and R.I. analyzed the data.

## Supporting information

Figure S1
