# Supplementary material for: A randomized, double‐blind, placebo‐ and positive‐controlled crossover study of the effects of durlobactam on cardiac repolarization in healthy subjects
Source: Clin Transl Sci. 2021 May 2;14(4):1423–30. doi: 10.1111/cts.12991 (PMC8301544; doi:10.1111/cts.12991)

**Supplementary Material**

Model-predicted ΔΔQTcF (mean and 90% CI) and estimated placebo-adjusted ΔQTcF (mean and 90% CI) across deciles of moxifloxacin plasma concentrations and predicted ΔΔQTcF interval at geometric mean peak moxifloxacin concentrations (PK/QTc population).


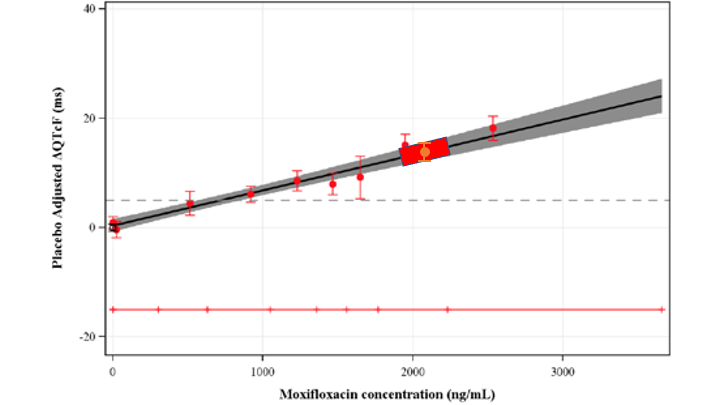

Supplement: Supplementary file 1 — Figure S1 [file CTS-14-1423-s001.docx]
